# All the Same? The Secret Life of Prion Strains within Their Target Cells

**DOI:** 10.3390/v11040334

**Published:** 2019-04-09

**Authors:** Ina M. Vorberg

**Affiliations:** 1 German Center for Neurodegenerative Diseases (DZNE e.V.), Sigmund-Freud-Strasse 27, 53127 Bonn, Germany; ina.vorberg@dzne.de; 2 Rheinische Friedrich-Wilhelms-Universität Bonn, 53127 Bonn, Germany

**Keywords:** Prion, transmissible spongiform encephalopathy, strain, PrP, endocytic trafficking

## Abstract

Prions are infectious β-sheet-rich protein aggregates composed of misfolded prion protein (PrP^Sc^) that do not possess coding nucleic acid. Prions replicate by recruiting and converting normal cellular PrP^C^ into infectious isoforms. In the same host species, prion strains target distinct brain regions and cause different disease phenotypes. Prion strains are associated with biophysically distinct PrP^Sc^ conformers, suggesting that strain properties are enciphered within alternative PrP^Sc^ quaternary structures. So far it is unknown how prion strains target specific cells and initiate productive infections. Deeper mechanistic insight into the prion life cycle came from cell lines permissive to a range of different prion strains. Still, it is unknown why certain cell lines are refractory to infection by one strain but permissive to another. While pharmacologic and genetic manipulations revealed subcellular compartments involved in prion replication, little is known about strain-specific requirements for endocytic trafficking pathways. This review summarizes our knowledge on how prions replicate within their target cells and on strain-specific differences in prion cell biology.

## 1. Introduction

Transmissible spongiform encephalopathies (TSEs) are devastating neurodegenerative diseases that are caused by prions, unconventional infectious agents composed of the aberrantly folded host-encoded prion protein PrP. Prions replicate predominately in the central nervous system (CNS) and lymphoreticular system. In the CNS, the prion particle PrP^Sc^ is mainly found associated with neurons and astrocytes [1]. Also, microglia stain positive for PrP^Sc^, likely because they internalize extracellular PrP^Sc^ [1]. Follicular dendritic cells are required for prion replication in the spleen [2]. PrP^Sc^ can also be found in peripheral nerves, placenta, gut, muscle, and other organs [3,4,5]. Inflamed non-lymphoid tissue has also been shown to accumulate prions under inflammatory conditions [6].

PrP^C^, the precursor of the disease-associated PrP^Sc^, is expressed mainly on neurons and astrocytes, but also other cell types [7]. PrP^C^ is a glycosylated, sialylated protein that is anchored to the cell membrane by a glycosyl-phosphatidyl-inositol (GPI) anchor. Prions multiply via a template-assisted process in which a PrP^Sc^ polymer binds to PrP^C^, thereby triggering its conformational switch to a β-sheet rich isoform that becomes part of the growing aggregate. Secondary nucleation events, such as fragmentation of PrP^Sc^ multimers, subsequently lead to the generation of infectious seeds that continue to recruit and convert PrP^C^ [8]. The three-dimensional fold of PrP^Sc^ polymers has so far not been resolved, but recent models propose a parallel in-register β-strand fold [9,10] or a three- or four-rung β-solenoid structure [11].

An interesting feature of prions is that they exist as strains with different biological properties. Prions strains have been originally identified upon transmission of field isolates to small ruminants and laboratory rodents [12]. Prion strains differ in incubation times, their transmissibility to other species, and are associated with strain-specific PrP^Sc^ distribution patterns and neurodegenerative changes in distinct brain regions [13]. Prion strains with different biological properties in inbred mouse lines were isolated through serial transmission of the SSBP/1 scrapie brain pool to small ruminants and rodents. Mouse-adapted prion strain 22L has been isolated upon transmission to mice [14] while strains 79A and 139A were derived from the same SSBP/1 passaged through goats and mice [12]. Strain Chandler was originally isolated by serially passaging prions from a goat source through mice [15]. The strain was later transferred to the Rocky Mountain Laboratories, where it was renamed RML [16]. We refer to this strain as Chandler/RML. Strain ME7 was derived from a Suffolk sheep with natural scrapie. The strain was obtained through high-dilution cloning in mice [17]. Also, human prion strains have been successfully adapted to mice [18].

Comparative analysis of PrP^Sc^ deposition and neuropathological changes in inbred mice infected with mouse-adapted scrapie strains reveals strain-specific differences in spongiform degeneration, gliosis, and PrP^Sc^ deposition in specific brain regions [19]. Strains exhibit a remarkable host cell tropism, with particular strains preferentially targeting astrocytes, neurons, or both [20,21]. In mice infected with prion strain 22L, PrP^Sc^ mainly deposits in astroglia in several brain regions during early stages of infection. In the same mouse line, strain Chandler/RML PrP^Sc^ associates with astroglia in the thalamus and cortex, but also neurons and neuropil in the substantia nigra and the hypothalamus [21]. Light microscopic and ultrastructural studies demonstrate that PrP^Sc^ deposition can be intracellular and extracellular [1]. The intra- and extracellular distribution and morphology of PrP^Sc^ deposits depend on the prion strain and genetic background of the host [1]. Electron micrographs and histological examination of brain tissue from prion-infected mammals including humans revealed prominent localization of disease-associated PrP in the interstitial space, on astrocytic processes, or on dendritic and somatic plasma membranes [1,22]. Disease-associated PrP on membranes was often associated with coated endocytic invaginations or extended, sometimes spirally twisted pit necks, as well as plasmalemmal microfolds. Intracellularly, pathological PrP was found within the endolysosomal system [1,22,23,24]. Importantly, comparative ultrastructural or immunohistochemical studies on the subcellular distribution of pathological PrP^Sc^ associated with mouse-adapted prion strains are lacking.

The existence of different prion strains has long posed a conundrum to the prion hypothesis that states that prions are composed of the misfolded host-encoded protein PrP [7]. As prion strains faithfully propagate their biological properties in the same host, strain-specific information must be enciphered independent of the PrP primary sequence. Indeed, PrP^Sc^ molecules associated with distinct prion strains differ in their posttranslational modifications such as glycosylation and sialylation patterns, their relative protease sensitivity, and their solubility in detergents [25]. It is now clear that PrP^C^ can adopt a range of diverse PrP^Sc^ conformations that differ in their physicochemical and biological properties [26]. The diversity of PrP^Sc^ structures likely provides the basis for the heritable properties of prion strains [26]. In vitro, strain-specific PrP^Sc^ amplification has been shown to depend on different cofactors such as RNA, phospholipids, or ganglioside GM1 [27,28,29]. These cofactors, however, are ubiquitously present in tissue, so it is unclear how they could contribute to strain diversity in vivo. Potential cofactors required for PrP^Sc^ amplification in vivo remain unknown.

Several hypotheses have been forwarded to explain the observed host cell tropism of prion strains. One possibility is that the transport of prion strains outside and/or inside the brain differs, resulting in different brain regions that become infected. Recent evidence does not support this scenario [30]. Alternatively, subpopulations of cells could express so-far unknown receptors that allow entry of only certain PrP^Sc^ conformers. Further, permissive cells might express specific cofactors or a subset of posttranslationally modified PrP^C^ molecules required for the replication of particular prion strains. Another possibility is that the intracellular fate of PrP^Sc^ particles associated with distinct strains differs when taken up by potential target cells. Here we summarize recent findings from cell culture models that point towards differences in cell biology for different prion strains.

## 2. Restricted Susceptibility of Cell Lines to Different Prion Strains

The mechanism of prion replication remained unknown for a long time. Early attempts to propagate prion infectivity in cell explants or permanent cell cultures were performed before PrP^Sc^ was identified as the main component of the infectious agent (reviewed by S.A. Priola [31]). Infection experiments revealed that the cellular requirements for the propagation of particular prion strains differ. An important finding here was that only a few PrP^C^ expressing cell lines can be infected with prions [32,33]. A major breakthrough was the detection of PrP^Sc^ in Chandler/RML-infected N2a neuroblastoma cells via immunoblot [34]. N2a subclones persistently infected with Chandler/RML soon became the major cell model to study the subcellular compartments involved in prion replication [35,36,37,38].

In vitro, neuronal and epithelial cell types, as well as microglia, fibroblasts, pancreatic B cells, lymphoblasts, myoblasts, and Schwann-like cells can support replication of selected prion strains (reviewed by S.A. Priola [31]). Cell lines that are particularly permissive to prions include the microglia-like cell line SMB [39], the hypothalamic neuronal cell line GT1 [40], and catecholaminergic neuronal CAD cells [41], but also kidney epithelial cell line RK13 [42] and fibroblast line L929 [43] (Table 1). While prion replication is usually studied in cells infected with mouse-adapted scrapie strains, murine cells or rabbit RK13 kidney epithelial cells ectopically expressing the PrP transgene of interest can also support replication of some mouse-adapted prion strains of human or bovine origin [33,41,44]. Importantly, few cell lines so far have been identified that can be infected with prions that have not been adapted to mice. RK13 cells engineered to express ovine or vole PrP were found to be permissive to some scrapie field isolates [45]. Likewise, RK13 cells stably expressing elk PrP can propagate chronic wasting disease prions [46]. The Madin–Darby–Bovine kidney cell line (MDBK) ectopically expressing bovine PrP was found to be susceptible to cattle-derived bovine spongiform encephalopathy (BSE) [47]. So far, no cell line has been identified that can propagate prions isolated directly from human brain. Attempts to infect RK13 cells exogenously expressing human PrP with sporadic Creutzfeldt-Jakob disease (CJD) prions were unsuccessful [48]. However, upon infection of wildtype mice with the same isolate, the resulting strain M1000 replicated in RK13 cells engineered to express mouse PrP [48] (Table 1). Prior passage of CJD strains in mice also enabled infection of mouse GT-1 cells [44]. Primary cerebellar granule cells derived from transgenic mice overexpressing human PrP, however, successfully propagated patient-derived sporadic, variant, and iatrogenic CJD, demonstrating that the mouse cell environment can support replication of human prions [49]. Very recently, first successful transmission of sporadic and variant CJD prions from human brain to astrocytes derived from human pluripotent stem cells was reported [50].

Surprisingly, PrP^C^ expressed by cell lines that are refractory to certain prion strains can be efficiently converted to PrP^Sc^ by the same strains in extracellular systems. When lysate of N2a cells expressing antibody-epitope tagged mouse PrP^C^ was incubated with brain homogenate from mice infected with different scrapie strains, all strains efficiently converted PrP^C^ to its protease-resistant isoform, even though cells are refractory to chronic infection with the strains ME7 and 87V tested [51] (Table 1). Likewise, PrP^C^ expressed by a cell line resistant to 87V was readily converted to PrP^Sc^ by the same strain in a cell-free conversion assay [52]. This suggests that the observed resistance was due to additional factors or cellular processes. The variety of different cell types susceptible in vitro demonstrates that potential cofactors involved in prion replication are also expressed by cells that are not main targets of prions in vivo. Interestingly, a particular cell culture can support the replication of diverse prion strains that maintain their strain properties when passaged back in inbred mice [44]. In line with this, co-infection studies in rodents demonstrate the competition of prion strains for cellular resources, suggesting that at least some strains target the same subpopulations of cells also in vivo [30]. Thus, cells can contain cofactors that support faithful replication of different prion strains, arguing against the hypothesis that cell-type specific expression of cofactors drives host cell tropism of prion strains [53].

Even more surprisingly, identified cell lines can be remarkably restrictive when it comes to replication of certain prion strains. For example, mouse-adapted strain Chandler/RML can infect a variety of different cell lines that are also permissive to strain ME7; however, a cell line that can propagate Chandler/RML prions can be refractory to infection with ME7 (Table 1) [31]. Also, subclones of permissive cell lines can be refractory to infection by a prion strain that replicates efficiently in the uncloned mother cell line [54,55]. Resistance was shown to be independent of PrP^C^ expression levels or PrP polymorphisms [56]. When subjected to in vitro amplification of PrP^Sc^ via protein folding cyclic amplification (PMCA), PrP^C^ from a resistant cell clone proved to be equally suitable as a substrate for Chandler/RML prions, arguing against the hypothesis that cell-type specific posttranslational modifications of PrP^C^ underly host cell tropism of prion strains [55]. Further analyses revealed that permissive and non-permissive N2a clones differed mainly in the expression of genes involved in the homeostasis of the extracellular matrix (ECM) [56]. The ECM comprises a dense network of extracellular macromolecules including collagens and glycoproteins such as proteoglycans. Interestingly, experimental downregulation of genes involved in sulfation of ECM resident heparan sulfate proteoglycans resulted in enhanced sequestration of PrP^C^ in the ECM and a concomitant increase in PrP^Sc^ in N2a cells infected with Chandler/RML [56]. How exactly the ECM contributes to PrP^Sc^ formation is unclear, but the fact that expression of ECM-regulating genes modulates prion infection demonstrates that factors other than PrP^C^ with specific cellular distribution can determine prion susceptibility.

As prions usually replicate without causing cytopathic effects in permanent cell lines, these in vitro models cannot be used to study toxic events associated with prion replication. Instead, neuropathologic changes can be observed in prion-infected primary neurons cultured for prolonged periods of time [65]. Difficulties in genetic and pharmacologic manipulation and cell type heterogeneity have so far limited the use of these cultures to study prion cell biology [66]. Permanent cell lines are therefore primarily used to study cell biological mechanisms of prion replication.

## 3. The Complex Organization of the Endocytic Pathway

**Figure 1:**
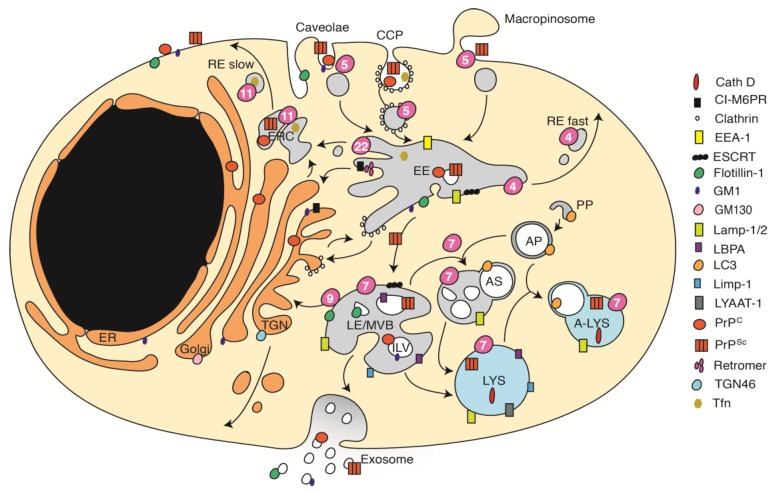
Endocytic vesicle trafficking pathways. Cargo is internalized through clathrin-coated pits (CCP), or clathrin-independent uptake routes via caveolae, rafts, flotillin-1/2 assisted pathways, or bulk uptake via macropinocytosis. Cargo is first sorted to early endosomes (EE), where it is disseminated to specific tubular subcompartments for fast recycling to the cell surface (RE fast), or slow recycling through recycling endosomes (RE slow) and the endocytic recycling compartment (ERC). Alternatively, cargo can be subject to retrograde transport to the trans-Golgi network (TGN). Cargo destined for degradation is trafficked to late endosomes/multivesicular bodies (LE/MVBs). LE/MVBs containing intraluminal vesicles (ILVs) can fuse with the plasma membrane to secrete cargo-loaded ILVs as exosomes. For cargo clearance, LE/MVBs fuse with lysosomes (LYS) or autophagosomes (AP) to form amphisomes (AS) that subsequently fuse with lysosomes. Autophagosomes can also directly fuse with lysosomes to form autolysosomes (A-LYS). Also, anterograde crosstalk between the TGN and endocytic pathway exists (not shown). Cellular locations of PrP^C^, PrP^Sc^, and marker proteins/lipids are indicated. RabGTPases, key regulators of intracellular vesicle trafficking, are numbered and symbolized by purple circles. Rab7a, Rab11a, and Rab22a regulate the dynamics of RE that emerge from EEs [86]. Cath D: Cathepsin D; LC3: lipidated form LC3-II; Tfn: Transferin. Note that the cellular distribution of marker proteins/lipids varies slightly for different cell types and in differentiated versus undifferentiated cells.

PrP^Sc^ formation involves conversion of PrP^C^ to its abnormal isoform. Consequently, cellular compartments that harbor PrP^C^ likely represent regions of initial isoform encounters and subsequent conversion processes. Research over the last few decades has demonstrated that the organization of the endocytic pathways is more complex than anticipated (Figure 1). Membrane proteins, lipids and cargo enter the endolysosomal system through clathrin-dependent and independent routes. Clathrin-dependent endocytosis (CME) involves the binding of ligands to specific receptors, which triggers the formation of clathrin-coated pits (CCPs). Clathrin-independent endocytosis (CIE) pathways are less well understood and comprise caveolae- or raft-mediated endocytosis, Flotillin-1-associated endocytosis routes and others. Rafts are specific microdomains highly enriched in cholesterol, sphingolipids and GPI-anchored proteins that serve as signaling platforms. Caveolae represent flask-shaped invaginations of a specific type of lipid raft that contains caveolin-1. Cell membrane associated PrP^C^ can be internalized via clathrin-, caveolin-, or raft-mediated pathways [67,68,69,70,71,72,73]. The fact that GPI-anchored proteins follow distinct endocytosis routes depending on the cell type likely accounts for differences observed in PrP^C^ internalization [74,75].

Independent of the internalization route, cargo-enriched endocytic carriers undergo fusion to form the early endosome (EE). EEs are highly dynamic, pleiomorph compartments that consist of a central vacuole (also termed sorting endosome, SE) with membrane invaginations, intraluminal vesicles (ILVs), and emanating thin tubular extensions [76,77]. EEs serve as sorting hubs that sort cargo for recycling to the cell surface and secretory pathway or through late endosomes/multivesicular bodies (LE/MVBs) to lysosomes for degradation. Vesicle trafficking and fusion are orchestrated by Ras-associated binding proteins (RabGTPases) and other regulatory proteins [78]. Cargo that directly recycles back to the cell surface is sorted into subdomains that subsequently form specialized transport carriers (fast recycling). Alternatively, cargo is first targeted to specialized endosomes, termed recycling endosomes (RE), that often cluster as a tubule-vesicular network in the perinuclear region, the so-called endocytic recycling compartment (ERC), for slow subsequent recycling back to the plasma membrane. EE and LE/MVBs also produce membrane-enclosed transport carriers for retrograde transfer of raft lipids, transmembrane proteins, and exogenously internalized cargo, such as cholera toxin or shiga toxin, to the secretory pathway [79]. Retrograde transfer from the EE to the trans-Golgi network (TGN) can either be direct or indirect through passage through the ERC [80]. Segregation of cargo into tubular subdomains for transport to specific subcellular compartments is orchestrated by specialized sorting machineries, such as the retromer complex, that are involved in cargo selection for retrieval or degradation [77]. Apart from retromer, at least three additional retriever multi-protein complexes have been identified that sort cargo away from lysosomal degradation [81]. Cargo destined for lysosomal degradation is sorted into ILVs as EEs mature into LE/MVBs. Sorting depends on the sequential action of different endosomal sorting complexes required for transport (ESCRT) that cluster ubiquitylated proteins on their way to the lysosome at degradative subdomains that eventually bud and release cargo-enriched ILVs into the lumen of EE and LE/MVBs [82]. Also ubiquitin-and ESCRT- independent sorting into ILVs exists. ESCRT and retrieval complexes, such as retromers, thus play antagonistic roles in cargo sorting and are segregated into distinct EE subdomains to ensure cargo sorting to either degradative or recycling pathways. LE/MVBs can subsequently fuse with lysosomes for cargo degradation. Alternatively, LE/MVBs can also fuse with the plasma membrane to release ILVs as exosomes into the extracellular space. At LE/MVBs, the endocytic pathway converges with the autophagic pathway, resulting in the fusion of LE/MVBs with autophagosomes, which are double-membraned structures that sequester cytosolic cargo for clearance. This fusion generates so-called amphisomes that subsequently fuse with lysosomes, forming the autolysosome. Autophagosomes can also directly fuse with lysosomes, adding another layer of complexity.

The precursor protein of the infectious prion particle is PrP^C^, a membrane protein tethered to the cell surface via a glycosyl-phosphatidyl-inositol moiety. Following synthesis of the polypeptide chain in the rough ER and attachment of complex glycans at two asparagnine-linked glycosylation sites in ER and Golgi, PrP^C^ reaches the outer surface of the cell membrane where it resides in lipid rafts [83]. Upon endocytosis, PrP^C^ is trafficked through EE and trafficked through the ERC back to the cell surface or alternatively to endolysosomal compartments for degradation. Chase experiments with antibodies binding to cell surface PrP^C^ argue that in N2a cells, PrP^C^ reaches EEs within 30 min and is subsequently transported to LE/MVBs within 2 h. Colocalization with lysosomal marker LYAAT-1 was not observed, potentially because PrP^C^ can be rapidly degraded by proteases [84]. Importantly, the subcellular distribution of PrP^C^ differs for different cell types. PrP^C^ almost exclusively resides on the plasma membrane of N2a cells, while in GT-1 cells, 50% of PrP^C^ can be found within subcellular compartments, including Golgi, EE, and ERC [85].

## 4. Involvement of the Endocytic Trafficking Pathway in Prion Biogenesis

First insights into the subcellular compartments involved in PrP^Sc^ replication came from biochemical assays using permanent cell lines persistently infected with mouse-adapted scrapie. In N2a cells chronically infected with Chandler/RML, enzymatic removal of PrP^C^ from the cell surface demonstrated that PrP^C^ first traversed the cell surface before being converted to PrP^Sc^ [35,87]. Only a fraction of PrP^Sc^ could be labeled with cell membrane-impermeable biotin, arguing that the majority of PrP^Sc^ was located inside the cell [35,67]. The presence of PrP^Sc^ in detergent-resistant cell lysate fractions containing ganglioside GM1 and H-ras suggested that some PrP^Sc^ was residing in subdomains that resemble rafts and caveolae [67]. Metabolic pulse-chase experiments revealed that labeled PrP^C^ acquired protease-resistance within 2 h with a t_1/2_ of approx. 3 h [35,87]. Even after prolonged chase, only approx. 5–10 % of PrP^C^ was converted into its proteinase K resistant isoform [35,87]. Endocytosis of PrP^C^ preceded formation of PrP^Sc^ in the same cellular system [35]. PrP^Sc^ was shown to be aminoterminally truncated within acidic organelles [36,88]. Inhibition of lysosomal hydrolases increased PrP^Sc^ accumulation, suggesting that lysosomes were sites of prion degradation [89]. Inhibition of endocytosis by cooling cells to 18 °C suggested that the endocytic pathway was not only a pathway required for PrP^Sc^ degradation but was actively involved in PrP^Sc^ formation [35].

While replication of conventional intracellular pathogens can be tracked by visualization of synthesized gene products that are normally absent from the cell, prion detection requires discrimination of normal and disease-specific PrP isoforms. The lack of antibodies that selectively bind to PrP^Sc^ in cells poses a major obstacle to microscopic analyses of prion-infected cells. A novel staining protocol that included harsh denaturants enhanced PrP^Sc^ antigenicity and allowed detection of pathologic PrP with minimal background levels of PrP^C^ [90]. For simplicity, we refer to both abnormal PrP detected following denaturation and proteinase K resistant PrP detected by immunoblot as PrP^Sc^. Confocal and ultrastructural analyses of Chandler/RML-infected N2a cells demonstrated that PrP^Sc^ was located primarily intracellularly in a vesicle-rich perinuclear area and lysosomes [37,90]. The immunocytochemical identification of subcellular compartments in which PrP^Sc^ resides relies on the colocalization of denatured PrP^Sc^ with proteins that are abundant in specific subcellular compartments, such as EEA-1 for EE, Lamp-1 for LE/MVBs and lysosomes, and Rab11 or internalized transferin (Tfn) for the ERC (Figure 1). However, markers are not exclusively present in specific subcellular sites and can also been found in other subcompartments as they are involved in intra-organelle shuttling of protein and lipids. This is especially important when it comes to the discrimination of compartments of the degradative pathway, because antibodies do not reliably discriminate LE/MVBs from lysosomes [91].

**Figure 2:**
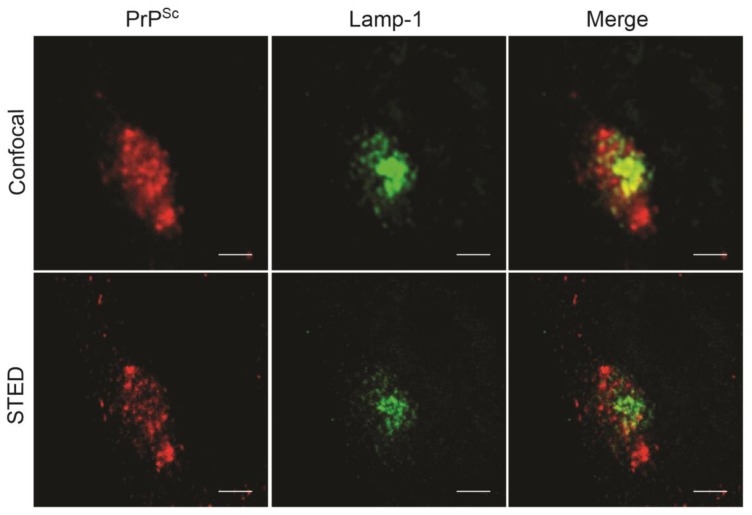
Comparison of spatial resolution of confocal microscopy and super resolution microscopy. N2a cells persistently infected with 22L were fixed and permeabilized, and antigens were denatured using 6 M guanidine hydrochloride. PrP^Sc^ was detected using antibody 4H11 [97]. Lamp-1 was detected using rabbit polyclonal antibody ab24170 (Abcam). Scale bar: 20 μm. Images were taken using the stimulated emission depletion (STED) microscope Leica TCS SP8 STED and a 93× objective. A single plane is shown. STED microscopy provides better spatial resolution compared to conventional confocal microscopy (images courtesy of Valerio Bonaldo, Vorberg laboratory).

A further problem when quantitatively analyzing cellular locations of PrP^Sc^ deposition is that laser scanning confocal microscopes or deconvolution processes suffer from limited spatial resolution of objects less than 200 nm apart [92]. This poses a problem when resolving signals from vesicles and compartments that cluster in the perinuclear cloud (Figure 2) [93,94]. Smaller differences in subcellular localization might be hard to dissect and would require discrimination of subdomains of the highly dynamic compartments such as ERC and LE/MVBs.

While recent studies that aim at elucidating prion biogenesis within cells usually include at least two prion strains, in many instances, strains are studied in different cellular models [84,85]. Importantly, there is substantial heterogeneity in the relative abundance of PrP^Sc^ in subcellular compartments of different cellular models even when infected with the same strain [84,95,96]. So far, comprehensive quantitative comparisons of the PrP^Sc^ distribution in the same cell line infected with different prion strains have not been performed. These limitations need to be kept in mind when trying to define the precise subcellular distribution of PrP^Sc^. However, colocalization studies and biochemical analyses show that the subcellular sites of PrP^Sc^ biogenesis and deposition in prion-infected cells broadly overlap with subcellular compartments that stain positive for disease-associated PrP in vivo.

## 5. Subcellular Distribution of PrP^Sc^ in Cells Infected with Different Prion Strains

Despite decades of research, it is still unclear whether the subcellular sites of replication differ for different prion strains in the same cell model. Comparison of different studies reveals that compartments of PrP^Sc^ deposition in N2a cells are somewhat similar for strains 22L and Chandler/RML (Table 2). The similarity of PrP^Sc^ deposition sites and the often identical effects that genetic or pharmacologic manipulations have on PrP^Sc^ accumulation in different cellular models [85,89] might be one reason why several studies do not even reveal the prion strain that replicates in their cell model [90,95,98,99]. In Chandler/RML-infected N2a cells, only a smaller fraction of PrP^Sc^ was detected on the plasma membrane using confocal microscopy [100,101]. Recent findings argue that the amount of PrP^Sc^ on the cell surface and associated with the ECM is underestimated [56]. PrP^Sc^ resided as strings and webs in GM1-positive regions on the cell surface [101]. Interestingly, strings appeared to also contain aminoterminally trimmed PrP^Sc^ molecules, suggesting that these molecules were either truncated on the cell surface within strings or were trimmed intracellularly and subsequently recycled back to the cell surface [101]. In N2a cells, Chandler/RML PrP^Sc^ fractions were associated with Lamp-1-positive compartments (Table 2) [84,100]. Hardly any staining was detected in LYAAT-1-positive vesicles. Some Chandler/RML PrP^Sc^ was also found in vesicles containing Rab9 [102]. Controversy exists regarding the presence of Chandler/RML PrP^Sc^ in EEs [84,102]. Chandler/RML PrP^Sc^ appeared to be prominent in the ERC according to co-staining with Tfn and Rab11 [102]. Rare staining was also observed in Golgi and TGN of N2a cells [102].

In N2a cells persistently infected with 22L, some PrP^Sc^ was detected on the cell surface [85,103,104]. PrP^Sc^ was found in clathrin-coated pits [103]. Some colocalization was also found with EE (Rab4, Rab5, EEA-1) [103,104]. Intracellularly, PrP^Sc^ was mainly found associated with Tfn-positive compartments [85]. 22L PrP^Sc^ partially colocalized with the retromer and the clathrin complex that coordinate cargo transport from different EE subdomains to the TGN [105]. The presence of 22L PrP^Sc^ in Golgi and TGN is unclear [85,102]. PrP^Sc^ was also present in Lamp-1-, LBPA-, or Rab7-positive compartments (markers for LE/MVBs and/or lysosomes) [85,95,103,104]. Slight differences in PrP^Sc^ colocalization with Flotillin-1 have been reported for strains Chandler/RML and 22L. Yamasaki et al. [104] showed intense colocalization of 22L PrP^Sc^ in Flotillin-1-positive intracellular compartments, while only minor colocalization was observed for strain Chandler/RML in the same cell line [84]. In summary, 22L and Chandler/RML PrP^Sc^ were mainly found in compartments staining for markers of EEs (EEA-1), ERC (Tfn, Rab11), and MVBs/lysosomes (Rab7, Lamp-1) in chronically infected N2a cells. PrP^Sc^ was also found on the plasma membrane and occasionally in Golgi and TGN. Of note, independent studies using a cell line infected with the same scrapie strain do not always come to the same conclusions (Table 2). Reasons for this could be the use of different sublines, antibodies, and/or limited spatial resolution.

Importantly, the subcellular distribution of PrP^Sc^ associated with particular prion strains might differ depending on the cell type and between permanent and primary cells. The subcellular distribution of PrP^Sc^ was recently studied in mouse cerebellar granule neurons infected with strains 139A, 22L, or ME7 [65]. Little co-staining was found with EE marker EEA-1 or ER marker BIP for any strain. Interestingly, PrP^Sc^ associated with strain ME7 was predominately found in the Golgi (marker Giantin) and Cathepsin D-positive compartments, highest levels of 139A PrP^Sc^ associated with Lamp-2 or Cathepsin D-positive vesicles. 22L PrP^Sc^ colocalized mainly with Giantin and Lamp-2. The observed differences in intracellular distribution could point to slight strain-dependent differences in PrP^Sc^ trafficking through the endocytic pathway.

## 6. Cellular Factors Involved in Prion Attachment and/or Uptake

Due to the usually low infection rates in permanent cell lines, insights into the cell biology of prion propagation have been mainly obtained from studies using chronically infected cell lines that were established via clonal selection of infected cells [34]. Establishment of subclones of prion permissive cell lines and novel cell models have now paved the way to also study the initial events of prion infection in more detail [41,42,43,54,59,73]. Just like viral infections, prion infections proceed through the individual steps of attachment of exogenous infectious particles and subsequent internalization followed by an establishment of infection (reviewed in Grassmann et al. [32]). PrP^Sc^ associated with different prion strains has different physicochemical properties that might impact interactions with cellular membranes or receptors. Independent of cell line and prion strain, cells that lack PrP^C^ can efficiently internalize PrP^Sc^, demonstrating that PrP^C^ does not serve as an exclusive receptor that mediates prion particle entry [32,42,61,73,107,108]. Several potential receptors for prions have been proposed, including the laminin receptor precursor, Lrp1, or glycosaminoglycans, such as heparan sulfate, but their role in the uptake of diverse prion strains is unclear [109,110,111]. At least in the case of glycosaminoglycans, mutant cell lines that lack these factors can efficiently internalize PrP^Sc^ [112]. Thus, to date, there is no evidence for any specific uptake receptor for prions, let alone for different receptors for different prion strains.

## 7. Establishment of Productive Infections

Even though strain-specific receptors for PrP^Sc^ internalization have not been identified, internalization rates can differ for different strains [42]. Differences in uptake could be due to different receptor engagements or different uptake pathways dependent on particle structure or size. Several studies demonstrated that purified, highly aggregated PrP^Sc^ enters the cell at lower speed [61,107,111]. However, while the aggregation state of PrP^Sc^ can be influenced by the purification procedure, a specific particle size range is also an intrinsic property of different prion strains [113,114].

How prion strains invade their target cells for productive infection is poorly understood. Macropinocytosis has been suggested as a pathway for uptake of PrP^Sc^ by primary neurons [115]. So far, it is unknown if this pathway also leads to productive infection. Experiments in different cell models using several prion strains have demonstrated that the majority of internalized PrP^Sc^ is routed to the lysosome for degradation [116,117,118,119]. At least some infectious particles must escape the cellular clearance machinery to establish persistent infection. Theoretically, differences in subcompartmental retention or intracellular trafficking of PrP^Sc^ associated with different strains could drastically affect the ability of a strain to establish persistent infection. It is possible that uptake by specific endocytic routes during prion exposure directs prion particles to pathways that are non-productive for certain strains. Indirect evidence for such a scenario comes from experiments with mouse cell line CF10 engineered to express antibody epitope-tagged mouse PrP that was exposed to scrapie strains 22L and 87V [52]. While PrP^Sc^ associated with both strains was rapidly taken up, strain 87V failed to establish persistent infection. Cell-free conversion experiments proved that 87V converted PrP^C^ expressed by CF10 cells more efficiently than 22L. However, once internalized, 87V PrP^Sc^ was much more rapidly disaggregated into smaller aggregates, despite the fact that its relative conformational stability determined in vitro was higher than that of 22L PrP^Sc^ [52]. The reason for the rapid disaggregation of 87V PrP^Sc^ is unclear. It is tempting to speculate that 87V PrP^Sc^ was delivered more efficiently to lysosomes, while at least a fraction of 22L PrP^Sc^ was able to escape or delay its route to disaggregation and degradation.

Recently, we showed that blockage of clathrin- or caveolin-1- (Cav-1) mediated endocytosis or macropinocytosis had only minor effects on 22L or RML PrP^Sc^ uptake by L929 cells permissive to both strains [73]. While the number of cells that had internalized 22L PrP^Sc^ remained relatively unchanged upon silencing of the clathrin heavy chain (CHC) or Cav-1, the total internalized PrP^Sc^ and the number and size of PrP^Sc^ puncta per cell increased. Fewer effects were observed for the uptake of Chandler/RML PrP^Sc^ by L929 cells partially depleted of Cav-1 or CHC. Thus, 22L and Chandler/RML PrP^Sc^ enter L929 cells predominately via clathrin- and caveolin-independent pathways. Pharmacologic inhibition of macropinocytosis did not reduce the number of cells that took up 22L or Chandler/RML PrP^Sc^, but slightly reduced number and size of intracellular 22L PrP^Sc^ puncta. A possible explanation for the inability to block PrP^Sc^ uptake is that prions have the ability to engage multiple internalization pathways for cell invasion. Alternatively, prions preferentially use alternative endocytosis routes to gain access to the cell [73]. However, the observed differences in the amount of PrP^Sc^ internalized by L929 cells argue that slight strain-dependent differences exist.

Surprisingly, while downregulation of CHC and Cav-1 had no drastic effects on the internalization of 22L and Chandler/RML PrP^Sc^, it strongly affected the establishment of productive infection in L929 fibroblasts. Western blot analysis of cells several cell doublings post prion exposure demonstrated that silencing of Cav-1 had no effect on the establishment of infections, arguing that caveolae or Cav-1-mediated trafficking was not required for the initial events of infection. By contrast, downregulation of clathrin component CHC strongly affected the establishment of infections in a strain-dependent manner. Silencing of CHC increased 22L infection, suggesting that clathrin-mediated pathways were not involved in the establishment of infection by strain 22L. By contrast, reduction of CHC decreased infection with strain RML, arguing that clathrin-dependent vesicle trafficking was required for the establishment of productive infection by strain RML [73]. One possible explanation for the finding that the establishment of productive infection depended on different endocytotic trafficking pathways is that internalized PrP^Sc^ could be trafficked through subcellular compartments that are either favorable or less favorable for a particular prion strain. Subcellular compartments could provide cofactors or specific environments essential for initial events in prion replication. Alternative trafficking through the endocytic pathway could limit or enhance access to these cofactors or conditions.

## 8. First Sites of PrP^Sc^ Formation during Acute Infection

Initial conversion of PrP^C^ to PrP^Sc^ appears to be a relatively fast process that occurs within minutes to hours following exposure to exogenous prion particles [61,108,120,121]. N2a cells expressing epitope-tagged PrP exposed to RML brain homogenate formed PrP^Sc^ rapidly on the cell surface within 1 min [108,120]. Initial cell surface PrP^Sc^ formation did not require endocytosis, as blockage of cargo internalization by cooling cells to 4 °C did not inhibit de novo PrP^Sc^ formation [108]. Newly formed PrP^Sc^ was subsequently transported to EEA-1-containing endosomes, Rab11-positive compartments, and later TGN46 and GM130-positive compartments, suggesting it was retrogradely transported to the Golgi. A small fraction of PrP^Sc^ was also found within Lamp-1- positive compartments.

Likewise, fluorescently-labeled, PK-treated 22L PrP^Sc^ entered N2a cells within minutes and was transported to a perinuclear region [122]. Exogenous PrP^Sc^ was identified in both the endocytic-recycling pathway colocalizing with labeled Tfn and the endo-lysosomal pathway colocalizing with labeled LDL, with the majority being present in the degradative pathway 30 h post inoculation. Newly formed PrP^Sc^ was observed first approximately 24 h post inoculation and increased over the next 36 h hours, while dye-labeled PrP^Sc^ steadily decreased to nearly undetectable levels. Newly formed PrP^Sc^ was first found on the plasma membrane (27%) and colocalized with EEA-1 (15%), Rab7a (30%), but rarely with Rab11 (5%) or Cathepsin D (3%). Importantly, even when PrP^Sc^ formation is observed within the first days post prion exposure, infection can be abortive, suggesting that downstream events determine the establishment of productive infections [121].

## 9. Acute and Chronic Prion Infections Depend on Different Intracellular Trafficking Processes

The findings that the majority of PrP^Sc^ is located intracellularly in permanent cells and that functional interference with endocytic trafficking pathways can reduce PrP^Sc^ accumulation suggests that prions undergo an intracellular life cycle for effective propagation. Recent findings argue that PrP^Sc^ formation during acute and chronic prion infection does not depend on the exact same trafficking pathways within the cell. We demonstrated that genetic manipulation of clathrin- or caveolin-mediated processes had different effects on prion biogenesis in acutely and chronically infected L929 fibroblasts [73]. As discussed earlier, in L929 fibroblasts caveolae or Cav-1 mediated processes were dispensable for the establishment of productive infections with strains 22L and Chandler/RML. However, silencing of Cav-1 in chronically infected cells decreased PrP^Sc^ accumulation independent of the prion strain. This is consistent with earlier findings in Chandler/RML-infected N2a cells, where Cav-1 overexpression enhanced PrP^Sc^ accumulation [123]. Further support for the hypothesis that cellular compartments of prion biogenesis differ during acute and chronic infection comes from studies with 22L-exposed N2a cells [104]. Newly formed PrP^Sc^ gradually shifted from plasma membrane, EE and LE/MVBs towards EE and RE during 72 h, suggesting that upon established infection, 22L PrP^Sc^ formation occurs in the endocytic-recycling pathway of N2a cells [104]. Interesting findings were also made with the cationic amphipilic drug U18666A, a compound known to inhibit egress of cholesterol from LE/MVBs and lysosomes. Treatment of N2a cells chronically infected with Chandler/RML led to decreased PrP^Sc^ accumulation [89]. Confocal microscopy analyses revealed that the same drug initially diminished colocalization of PrP^Sc^ with EEA-1 and increased its association with Lamp-1-positive vesicles, suggesting that enhanced transport of PrP^Sc^ to the degradative pathway was the reason for prion loss in chronically infected N2a cells [106]. Surprisingly, presence of the same drug during the first 24 h of infection had no influence on the establishment of persistent infections with strains 22L and RML, at least when assessed 1–2 weeks post infection. This treatment had no or only marginal effects on cell surface localization, raft residency or endocytosis rates of PrP^C^ [89,106]. Thus, increased PrP^Sc^ transport to the degradative pathway did not critically affect early events of prion infection. Chronically infected cells appeared to be more sensitive to redistribution of PrP^Sc^ to the degradative pathway. Thus, while the majority of exogenous PrP^Sc^ could have been transported to LE/MVBs and lysosomes, a fraction of PrP^Sc^ was apparently able to escape degradation and initiate conversion in upstream compartments, such as the cell surface or the ERC.

## 10. Subcellular Sites of PrP^Sc^ Formation in Chronically Infected Cells

Functional interference assays that block specific vesicle trafficking processes have shed some light onto possible compartments of intracellular PrP^Sc^ formation. It needs to be kept in mind that chemicals often also perturb other parts of the secretory and endocytic pathways. For example, Brefeldin A blocks protein exit from the ER and Golgi but also induces tubulation of the ERC and changes in the endolysosomal system [124,125]. Similar difficulties can arise when genetic approaches are used to impair intracellular endocytic trafficking processes. A Rab11 mutant for example not only interferes with cargo recycling through the ERC but also affects autophagosome biogenesis [126]. Likewise, inhibition of the retromer complex impairs shuttling of specific cargo from EE to the ERC and plasma membrane but can also negatively affect lysosomal proteolytic activity and autophagy [127]. As drugs and genetic manipulations of vesicle trafficking can have different phenotypic effects on cell lines and the distribution of marker proteins, meticulous characterization of pathway perturbations is required to draw meaningful conclusions [124,128]. This can be accomplished by testing the effect of the manipulation on the trafficking of well-characterized cargo such as labeled Tfn, cholera toxin, shiga toxin, or dextran [73,85]. Pharmacologic or genetic manipulation of vesicle trafficking also alters the concentration and subcellular distribution of lipids [95,129] that act as cofactors for PrP^Sc^ formation in vitro [27,28,29]. These limitations likely also affect the results obtained in different cellular models and need to be kept in mind when interpreting results of genetic manipulations (see below). Studies that include functional assays with control cargo are listed in Table 3.

As genetic and pharmacologic manipulations can have different effects on different cell lines, results are here summarized for the cell lines GT1, SMB, and N2a. Earlier studies suggest that the ERC is the major site of PrP^Sc^ formation in GT1 cells persistently infected with Chandler/RML (Table 3) [85]. Expression of GFP-Rab4 N121I that impairs recycling from EE had no effect on PrP^Sc^ levels in RML-infected infected GT1, arguing that the fast recycling pathway was dispensable for prion biogenesis. Tagged versions of wildtype Rab22a or the constitutively active mutant Rab22a Q64L can cause the formation of enlarged EEA-1-positive endosomes [86,124]. Expression of GFP-Rab22a resulted in retention of Tfn in GFP-Rab22-decorated swollen EE, suggesting that Rab22a overexpression impaired cargo sorting from the EE to the ERC [124]. Similarly, overexpression of GFP-Rab22a caused retention of Tfn in enlarged EE in GT1 cells chronically infected with prion strain Chandler/RML [85]. Chandler/RML PrP^Sc^ was initially enriched in Rab22a-positive vesicles, but total levels were subsequently reduced. SiRNA mediated depletion of Rab22a also led to a reduction of PrP^Sc^ in Chandler/RML-infected GT1 cells [85]. Inhibition of lysosomal degradation demonstrated that Rab22a downregulation did not increase total PrP^Sc^ levels relative to control cells expressing Rab22a, arguing that formation and not the clearance of PrP^Sc^ were affected by the knock-down [85]. Rab22a was recently shown to regulate the dynamics of a subpopulation of Rab11-positive REs [86,124]. Rab22a localizes to endosomal buds on EE and initiates or extends tubular structures that subsequently undergo fission to form tubular RE [86]. Rab22a-positive RE likely represent a subpopulation of RE that emerges from EE subdomains spatially separated from retromer-dependent tubular endosomes [86]. These results argue that a recycling pathway, including a specific subset of Rab22a-dependent RE, is involved in PrP^Sc^ biogenesis. To interfere with the Rab11-dependent recycling pathway, a dominant negative Rab11 S25N mutant was expressed in Chandler/RML-infected GT1 cells. This did not interfere with transport of Tfn from EEs to the ERC, but delayed Tfn exit from ERC and impaired its recycling from the ERC to the PM [85]. This treatment increased PrP^Sc^ levels in a compartment staining positive for Tfn; however, total PrP^Sc^ levels only slightly increased. This suggested that recycling to the cell surface was not required for PrP^Sc^ formation [85].

A recent study by Yim et al. [95] in SMB cells chronically infected with prion strain 139A [39] proposed that LE/MVBs are actively involved in PrP^Sc^ formation (Table 3). Rab7a is a key regulator of endocytic trafficking that controls maturation of EE into LE/MVBs, fusion between LE/MVBs and lysosomes, and autophagic maturation [130,131,132,133]. Expression of the dominant negative mutant Rab7a T22N or silencing of Rab7a resulted in the initial accumulation of PrP^Sc^ in Lamp-1/cation-independent mannose 6 phosphate receptor (CI-M6PR) double-positive swollen endosomes and a subsequent drastic decrease of PrP^Sc^. Earlier studies had demonstrated that Rab7a blockage causes retention in CI-M6PR in EEs [128]. CI-M6PR is primarily located at the TGN and shuttles lysosomal enzymes from the TGN to EEs. Retrograde transport to the TGN is mediated by a retromer-dependent process [134,135]. Both retromer and Rab7a are involved in CI-M6PR sorting at the EE for transport to the TGN [136,137]. Depletion of retromer subunit Vps26 also resulted in Lamp-1/CI-M6PR double-positive endosomes but increased total PrP^Sc^ levels. Only a fraction of PrP^Sc^ was found associated with Lamp-1-positive endosomes. Thus, retromer-mediated transport from EE to TGN was not involved in 139A PrP^Sc^ formation in SMB cells. Interestingly, overexpression of GFP-Rab22a or its constitutively active mutant Rab22 Q64L caused swollen EEA-1/Lamp-1-positive endosomes. Similar to manipulations of Chandler/RML-infected GT1 cells [85], this treatment also caused a strong reduction of total PrP^Sc^ levels in SMB cells. As described earlier, Rab22a is involved in cargo sorting from the EE to the ERC and back to the cell surface, suggesting that inhibition of the recycling pathway decreased the PrP^Sc^ load. Unfortunately, no co-detection of PrP^Sc^ with Rab11 or Tfn was performed in 139A-infected SMB cells, so it is unclear if any of these manipulations caused transport of PrP^Sc^ to the ERC. A reduction in total PrP^Sc^ over time was observed when silencing Hrs and Tsg101, core components of ESCRT-0 and ESCRT-1, respectively. Both treatments also caused initial retention of PrP^Sc^ in swollen endosomes positive for EEA-1 and Lamp-1 and subsequent PrP^Sc^ loss. No costaining was performed for CI-M6PR, so it is unclear if the swollen endosomes were related to the ones observed upon Rab7a silencing. However, as ESCRT complexes are involved in LE/MVB maturation [138], authors concluded that the LE/MVB is actively involved in PrP^Sc^ formation. It needs to be noted that ESCRT complexes have recently been reported to already act upstream of LE/MVBs [139].

Studies in 22L-infected N2a cells argue that the fast Rab4-mediated recycling pathway is not involved in 22L prion biogenesis (Table 3) [85,140]. Silencing of core components of the retromer complex increased 22L PrP^Sc^ in N2a cells, suggesting that retrograde transport from the EE to the TGN is dispensable for 22L prion formation [95]. While silencing of components of the clathrin coat complex affected the subcellular distribution of PrP^Sc^, total levels were not affected, suggesting that clathrin-mediated processes were not required for prion biogenesis in N2a cells persistently infected with strain 22L [105]. Further studies demonstrated that a small fraction of PrP^Sc^ was associated with clathrin-coated vesicles in 22L-infected N2a cells. siRNA-mediated knockdown of ESCRT-0 complex component Hrs caused reduction of PrP^Sc^ after several days [95].

A similar reduction was observed when ESCRT-I complex protein Tsg101 was silenced in 22L-infected N2a [95], arguing that in 22L-infected N2a cells, maturation of MVBs is crucial for prion accumulation. Importantly, Marijanovic et al. [85] demonstrated that overexpression of GFP-Rab22a, shown to drastically reduced PrP^Sc^ accumulation in Chandler/RML-infected GT1 cells, had no effect on total PrP^Sc^ levels in 22L-infected N2a cells. Authors further demonstrated that expression of GFP-Rab22 was ineffective at inhibiting transfer of Tfn from the EE to the ERC. Thus, in N2a cells, manipulation of the Rab22a-regulated vesicle trafficking appeared to affect the recycling pathway downstream of the EE to ERC transport, implicating that the ERC can still be involved in PrP^Sc^ formation. It is unknown if silencing of Rab22a would have had similar effects. Further interesting experiments could include silencing of Rab11 to assess the role of Rab11-dependent recycling on PrP^Sc^ accumulation. In summary, while there appear to be differences in prion biogenesis that could relate to cell type or prion strain, there is general consent that efficient PrP^Sc^ formation requires functional endosomal trafficking along the recycling and/or degradative pathway.

## 11. Considerations for Future Research on Prion Cell Biology

Understanding prion strain-specific host cell interactions is critical for the development of effective therapeutic strategies. Substantial progress has been made in identifying subcellular compartments of PrP^Sc^ accumulation and in characterizing endocytic trafficking pathways involved in prion biogenesis. Dynamic subcellular cycling of PrP^Sc^ was shown to play an essential role in efficient PrP^Sc^ formation. The cell surface and ECM, the LE/MVBs, and/or the ERC likely represent subcellular compartments of PrP^Sc^ biogenesis. Extensive research over the last few decades has reshaped our view on endocytic cargo trafficking. Endosomal compartments are highly dynamic, and different macromolecular machineries control segregation of cargo into distinct subdomains for cargo transport to the retrieving/recycling or degradative pathway. To which extent distinct vesicular trafficking pathways are involved in strain-dependent PrP^Sc^ formation remains to be determined.

A major complication is that the performed studies relied on different cellular models or compared prion formation in different cell lines infected with distinct prion strains. As considerable differences exist in endocytic trafficking pathways among different cell types, such comparisons are not suited to reveal strain-specific differences in prion biogenesis. Future studies will ideally focus on well-characterized prion strains in cellular models that are easy to manipulate chemically and pharmacologically. Primary neuronal and astrocytic cultures reflect more accurately the in vivo situation but do not constitute homogeneous cell populations and are more difficult to genetically or chemically manipulate. When choosing a cell model, one should also take into account the genetic instability of permanent cell lines that strongly affects permissiveness of cell clones. Consequently, different expression profiles can also be expected in prion-infected cell populations that originate from the same cell line but have separate passage histories. As the cellular requirements differ during the establishment of infection and persistent infection, highly susceptible cell clones will help to identify how manipulations of endocytic trafficking pathways affect uptake of PrP^Sc^, the establishment of productive infections and PrP^Sc^ formation and/or degradation. The use of tagged PrP^Sc^ as inoculum or the expression of tagged PrP^C^ will further allow discrimination of inoculum from endogenous PrP isoforms. Ideally, those endocytic trafficking pathways should be manipulated genetically for the strongest effects on PrP^Sc^ accumulations that have been reported, such as traffic regulated by Rab7a, Rab11, or Rab22a. Concomitant functional assays should control for effects of manipulations on specific endosomal trafficking pathways, using well-characterized cargo such as dextran, cholera toxin, or Tfn. If possible, triple-detection for specific markers and cargo should be performed to more carefully characterize the subcompartments after genetic/pharmacologic manipulation. Finally, quantitative comparative studies by EM or super resolution microscopy are urgently needed to more accurately determine the subcellular distribution of PrP^Sc^ associated with different prion strains.

## Figures and Tables

**Table 1:** Cell lines commonly used in prion research.

Cell Line	Origin	Mouse-Adapted TSE Strain										References
		Scrapie					Other TSE					
		Ch./RML	79A	139A	22L	ME7		87V	Fu-1 ^b^	M1000 ^c^	301C ^d^	
N2a	Mouse neuroblastoma											[33,34,57,58]
SMB	Mouse brain cells											[39]
GT-1	Mouse hypothalamic neurons											[40,44,59]
CAD5	Mouse catecholaminergic neurons											[41,60]
SN56	Mouse septal neurons											[61,62]
L929	Mouse fibroblasts											[41,43,63]
RK13	Rabbit kidney epithelial (moPrP) ^a^											[42,64]

^a^ Cells genetically engineered to overexpress mouse PrP (MoPrP); ^b^ Strain Fu-1 was derived from a human Gerstmann–Sträussler–Scheinker case transmitted to mice; ^c^ M1000 was isolated upon transmission of sporadic Creutzfeldt-Jakob disease (CJD) to mice; ^d^ 301C was isolated upon inoculation of brain homogenate from a bovine spongiform encephalopathy case into mice. Green boxes symbolize susceptibility, orange boxes symbolize resistance. Note that in many cases, subclones were used for infections. TSE: transmissible spongiform encephalopathy.

**Table 2:** Cellular localization of PrP^Sc^.

Cell Line	Clone	Strain	PM	Flotillin-1	Clathrin	Caveolin-1	Giantin	TGN38	CI-M6PR	Rab4	Rab5	EEA-1	Tfn/Tfr	Rab11	Rab7	Rab9	Lamp-1	Limp-2	LYAAT-1	LBPA	Cathepsin D	LC3	Detection	Reference
**N2a**	H6	22L	+ ^c^									+ ^c^						+ ^c^					IF	[103] ^b^
	H6	22L			+ ^d^							+ ^c^						+					EM	[103]
		22L	+ ^b^				-					+	+++							+			IF	[85] ^e^
	3	22L	+	+++				(+)		+	+			+	+	+	++						IF	[104]
	3	22L										++	++				+++						IF	[106]
	C24 ^a^	22L	-				+	+				+	+++	++		++							IF	[102] ^c^
		22L							+			+					++						IF	[95] ^c^
	3	22L, 72 h	(+)									+++			+++						(+)		IF	[106]
	^a^	Ch./RML		-			-					-					+++		-				IF	[84] ^c^
		Ch./RML	++														+++				+		IF	[100] ^f^
	C24 ^a^	Ch./RML					+	+				++	+++	++		++							IF	[102] ^c^
		Ch./RML	++																				IF	[101] ^c^
**GT-1**		22L	++																				IF	[101] ^c^
	7	22L					(+)	(+)		+	+		++	++	++		+				+		IF	[105] ^c^
**GT1**	7	Ch./RML		++								-					+++		-				IF	[84] ^c^
		Ch./RML	+				-					+	++							+			IF	[85] ^e^
		Ch./RML															+					+	IF	[91] ^e^
		Ch./RML	++																				IF	[101] ^c^
**SMB**		139A	-						+			+					++						IF	[95] ^c^

^a^ Cells overexpressing mouse PrP; ^b^ majority PrP^Sc^ outside EEA-1 or Lamp-1-positive compartments; ^c^ not quantitative; ^d^ clathrin cages observed; ^e^ quantitative analysis; ^f^ quantitative analysis of PrP^Sc^ in the perinuclear region. Ch./RML: Chandler/RML; PM: plasma membrane; IF: immunofluorescence; EM: electron microscopy.

**Table 3:** Genetic manipulation of endoctyic trafficking and its effect on PrP^Sc^.

Cell Line	Strain	Manipulation	PrP^Sc^ Colocalization	Total PrP^Sc^	Observed Effect on Endosomes, Control Cargo	Ref.
**N2a**	Ch./RML	Rab4 S22N	n.d. *	Increase	n.d.	[140]
		Rab6 Q72L	n.d. *	Increase	n.d.	[140]
		Rab9 WT	n.d.	Reduction	n.d.	[89]
	22L	Rab4 S22N	n.d. *	Increase	n.d.	[140]
		Rab4 N121I	n.d.	No effect	n.d.	[85]
		Rab6 Q72L	n.d. *	Increase	n.d.	[140]
		Rab22a WT	n.d.	No effect	No inhibition of transport Tfn from EE to ERC	[85]
		siRNA Hrs	n.d.	Reduction	n.d.	[95,141]
		siRNA Tsg101	n.d.	Reduction	n.d.	[95]
		siRNA Clint-1	Redistribution PrP^Sc^ from Tfn-positive vesicles to Lamp-1-positive vesicles	No effect	n.d.	[105]
		siRNA Ap1g1	n.d.	Increase	n.d.	[105]
**GT1**	Ch./RML	Rab4 N121I	n.d.	No effect	n.d.	[85]
		Rab11 S25N	Higher PrP^Sc^ levels in cellular compartment positive for Tfn Rare PrP^Sc^ in LBPA-positive endosomes	Increase	No inhibition of transport Tfn from EE to ERC Impaired recycling Tfn from ERC to PM Strong colocalization Tfn with Rab11 S25N-GFP	[85]
		Rab22a WT	PrP enriched in EEA-1-positive endosomes (no denaturation step) No PrP^Sc^ in GFP-Rab22a-positive cells after 6 days	Reduction	Enlarged EEA-1/Rab22a-double positive endosomes Normal internalization of Tfn Tfn retained in GFP-Rab22a-positive endosomes	[85]
		siRNA Alix	Strong colocalization Tfn and PrP^Sc^	Increase	Less LBPA-positive endosomes Less lysosensor-positive endosomes	[85]
**SMB**	139A	Rab5 Q71L	Colocalization with EEA-1/Lamp-1 double-positive endosomes	Reduction	EEA-1/Lamp-1 double-positive endosomes	[95]
		Rab7a T22N	Colocalization with enlarged Lamp-1-positive endosomes	Reduction	Enlarged Lamp-1/CI-M6PR double-positive endosomes	
		Rab7a WT	No change in PrP^Sc^ distribution	No effect	No enlarged Lamp-1-positive endosomes	
		Rab7a Q67L	No change in PrP^Sc^ distribution	No effect	No enlarged Lamp-1-positive endosomes	
		Rab22a Q64L	n.d.	Reduction	Enlarged EEA-1/Lamp-1-double-positive endosomes	
		Rab22a WT	n.d.	Reduction	EEA-1/Lamp-1 double-positive endosomes	
		siRNA Hrs	Initial colocalization with EEA-1/Lamp-1-positive endosomes	Reduction	EEA-1/Lamp-1 double-positive endosomes	
		siRNA Tsg101	Initial colocalization with EEA-1/Lamp-1-positive endosomes	Reduction	EEA-1/Lamp-1 double-positive endosomes	
		siRNA AILIX	Unchanged colocalization with Lamp-1-positive endosomes	Increase	n.d.	
		siRNA SNX2 (retromer)	n.d.	Increase	n.d.	
		siRNA Vps26 (retromer)	Partial colocalization with enlarged Lamp-1/CI-M6PR endosomes	Increase	Enlarged Lamp-1/CI-M6PR double-positive endosomes	

* Confocal microscopy not performed. Total PrP^Sc^ was detected using immunoblotting. Ch./RML: Chandler/RML; EE: early endosomes; ERC: endocytic recycling compartment; PM: plasma membrane; n.d.: not done; siRNA: silencing RNA. Grey boxes mark overexpressed RabGTPase.
